# Tanfloc-Modified Titanium Surfaces: Optimizing Blood Coagulant Activity and Stem Cell Compatibility

**DOI:** 10.1021/acsbiomaterials.4c02106

**Published:** 2025-02-27

**Authors:** Ramesh Singh, Liszt Y. C. Madruga, Aniruddha Savargaonkar, Alessandro F. Martins, Matt J. Kipper, Ketul C. Popat

**Affiliations:** † Department of Bioengineering, College of Engineering and Computing, George Mason University, Fairfax, Virginia 22030, United States; ‡ Department of Mechanical Engineering, Colorado State University, Fort Collins, Colorado 80523, United States; § Department of Chemical and Biological Engineering, Colorado State University, Fort Collins, Colorado 80523, United States; ∥ Department of Chemistry, Pittsburgh State University, Pittsburgh, Kansas 66762, United States

**Keywords:** titania nanotube, surface functionalization, medical implants, protein adsorption, platelet activation

## Abstract

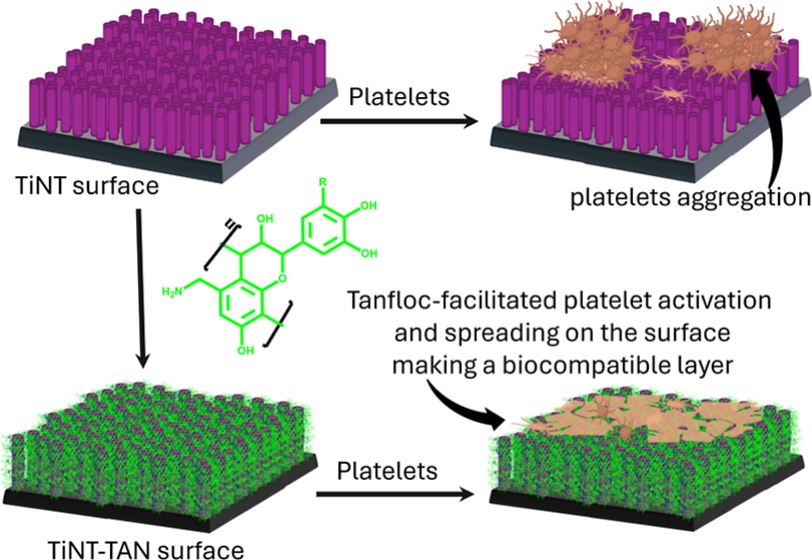

This study explores the synergistic effects of combining titania nanotubes (TiNTs) with the biopolymer Tanfloc (TAN) to enhance the surface properties of TiNTs for biomedical applications. We investigated the interactions of blood components and human adipose-derived stem cells (ADSCs) with TiNT surfaces covalently functionalized with Tanfloc (TAN), an aminolyzed polyphenolic tannin derivative. The functionalized surfaces (TiNT-TAN) have great potential to control protein adsorption and platelet adhesion and activation. Fluorescence and scanning electron microscopy (SEM) were used to analyze platelet adherence and activation. The amphoteric nature and multiple functional groups on TAN can control blood protein adsorption, platelet adhesion, and activation. Further, the modified surface supports adipose-derived stem cell (ADSC) viability, attachment, and growth without any cytotoxic effect. The TAN conjugation significantly (****p < 0.0001) increased the proliferation rate of ADSCs compared to the TiNT surfaces.

## Introduction

When a medical device is introduced into the body, its surface becomes the primary point of contact with the biological environment, initiating a complex series of interactions that precede the desired function.^1^ Physical and chemical surface properties such as roughness, morphology, charge, and chemical composition of a biomaterial determine the material’s behavior at the interface with biological systems and determine how the medical device integrates with the surrounding environment and is accepted by the body.^1−4^ These surface characteristics collectively shape the biomaterial’s performance and biocompatibility, ultimately affecting its success in medical applications.^2^

Hemocompatibility is a vital component of biocompatibility for biomaterials that contact blood, particularly in medical devices. It refers to a material’s capacity to interact with blood without inducing adverse effects such as hemolysis, thrombus formation, or activation of the complement system. Hemocompatibility evaluation is a complex process that examines various interactions between blood components and material surfaces.^5−7^ These interactions can initiate protein adsorption, platelet activation, and blood coagulation.^5,8^ Platelet activation plays a pivotal role in thrombus formation and can be either beneficial or detrimental depending on the specific medical application.^5,8−11^ Platelets contain alpha granules that store various growth factors. Upon platelet activation, these growth factors are released and promote tissue regeneration, wound healing, and the successful integration of medical devices by preventing blood loss and providing a matrix for tissue repair.^10−12^ In dental applications, titanium implants may achieve better osseointegration through platelet-mediated processes. Platelet activation can be advantageous in orthopedic procedures, including joint replacements, spinal fusion devices, and bone grafts.^13−15^ However, in cardiovascular applications, excessive thrombosis can lead to complications like vessel blockages, aneurysms, or cardiac arrest.^8,15^ Therefore, medical device surfaces must interact with blood in a controlled manner, ensuring optimal platelet activation and regulated blood clotting to balance healing promotion and the prevention of dangerous thrombosis.^8,10,15^

The superior biomechanical characteristics and exceptional corrosion resistance make titanium-based materials an optimal choice for biomedical devices.^16,17^ Various surface modification strategies, including nanostructure engineering, drugs, and biomaterial coatings, have been reported to improve the biological performance of titanium medical devices.^18^ Fabricating nanostructures such as titania nanotubes (TiNTs), nanopillars, nanopores, and nanostructured rough surfaces has improved biocompatibility.^19,20^ TiNTs offer unique advantages for controlling surface properties, including increased surface area, controllable size, and highly ordered surface arrangement.^21,22^ The fabrication parameters, such as anodization voltage, duration, electrolyte composition, and post-treatment conditions, play crucial roles in determining the final properties of TiNTs.^22,23^ For instance, the anodization voltage primarily affects nanotube diameter and length, while electrolyte composition influences the oxide formation and dissolution rate. Postanodization heat treatment can alter crystallinity, significantly impacting their performance in various applications.^21,24,25^ Studies have demonstrated that titania nanotubes’ morphology, structure, and wettability influence cellular functionality, including fibroblast adhesion, proliferation, differentiation, and decreased bacterial adhesion to a certain degree.^26−31^ The biomedical advantages of titania nanotubes are exclusively derived from their unique surface structure and topographical characteristics, not from any intrinsic material properties since they are biologically nonreactive.

Biofunctionalization of these nanostructures can further enhance physiological functions, such as bone tissue integration, antibacterial properties, and blood compatibility, depending on the nature of the functionalizing or coating material.^18,32−34^ However, most coating or surface modification methods target one specific issue, such as implant failure caused by infection, rather than promote a combined solution to address multiple biomaterial performance outcomes. To address these limitations, recent research has focused on developing multifunctional surfaces that simultaneously tackle multiple challenges.^13,18,35−37^ To overcome these limitations, the application of multifunctional biopolymers and combinations of various biopolymers and polyelectrolytes together with nanoengineered surfaces is of growing interest. Tanfloc is an amino-functionalized polyphenolic compound derived from condensed tannins, commonly used as an organic coagulant and flocculant.^38^ The amphoteric nature allows it to form polyelectrolyte complexes with various counter-polyelectrolytes and be used as a coating for biomaterials.^39^ Tanfloc alone and combined with polyelectrolytes such as heparin, glycosaminoglycans, alginate, and chitosan have demonstrated strong biocompatibility, antibacterial activity, and osteogenic differentiation with minimal toxicity toward mammalian cells.^39−42^ The combination of its amphoteric nature, polyphenolic structure, and ability to form electrostatic interactions makes Tanfloc a versatile and effective antibacterial agent.^39−41^ However, these biopolymer coatings are only physically absorbed or rely on weak, noncovalent interactions with the surface, which can compromise their long-term effectiveness as antibacterial agents may degrade or detach over time. A recent study has shown that covalently grafted Tanfloc on the titania nanotube (TiNT) surfaces without altering the nanotube array strongly inhibits bacterial adhesion and biofilm formation.^43^ Covalent conjugation provides sustainable functionality by combining the effects of nanostructures with the inherent antibacterial properties of Tanfloc. Therefore, combining the topographical effect of TiNT arrays, along with covalent conjugation of multifunctional, biodegradable, and biocompatible natural biopolymer Tanfloc, can enhance the compatibility and efficiency of the medical device.^43^ Thus, this study investigates vital aspects of the biological response on Tanfloc-modified titania nanotube biomaterial surfaces such as cellular adhesion, viability, growth, and blood interaction.

## Materials and Methods

### Materials

Thermo Fisher Scientific Chemicals Inc. provided diethylene glycol (DEG). 48% hydrofluoric acid (HF) was purchased from KMG Electronic Chemical. Oakwood Chemical provided the carboxyethyl silane-triol (CES) disodium salt 25% in water. We bought *N*-hydroxysuccinimide (NHS), 1-ethyl-3-(3-dimethylaminopropyl) carbodiimide hydrochloride (EDC), sodium monophosphate, and 2-morpholinoethanesulfonic acid (MES) from Sigma-Aldrich. Tanac SA (Montenegro-RS, Brazil) provided the Tanfloc (about 600 kDa).^69^

### Synthesis of Titania Nanotubes on a Titanium Surface

Titania nanotubes (TiNT) were synthesized on the surface of a commercially available medical-grade pure titanium sheet with a 0.5 mm thickness. The standard laboratory process of electrochemical anodization and annealing was employed to synthesize TiNT.^43,44^ In detail, the titanium sheet was sonicated in isopropyl alcohol and deionized water for 10 min and dried. Then, the cleaned Ti was subjected to anodization at 55 V in 95% diethylene glycol (DEG), 3% DI water, and 2% hydrofluoric acid-containing electrolyte solution for 22 h. The anodized surface was then rinsed with water and isopropyl alcohol and dried, followed by annealing at 530 °C for 3 h with a 15 °C/min temperature increment.

### Tanfloc Conjugation

The covalent conjugation of Tanfloc was done using the previously reported protocol.^43^ Titania nanotube sheets were immersed in a solution of carboxyethylsilanetriol (CES)-disodium salt dissolved in a phosphate buffer solution for 5 h at room temperature and kept in a shaker to allow the reaction to proceed. After the reaction, the sheet was washed with phosphate buffer followed by 2-morpholinoethanesulfonic acid (MES) buffer to remove any nonlinked carboxyethylsilanetriol (CES) from the surface.^48,49^ The CES-linked titania nanotube sheet was immersed in the EDC/NHS solution and activated for 2 h on a shaker. Ten mg/mL of Tanfloc in DMF was sonicated for 15 min to create a homogeneous suspension in a separate beaker. Then, the NHS-activated titania nanotube surface was immersed to allow the conjugation of Tanfloc via amide coupling.^48,50,51,72^ After the reaction, the sheet was washed with solvent to remove unreacted Tanfloc from the surface and then dried for further use.

### Contact Angle Measurement

The water contact angle on different surfaces was calculated through a Ramé-Hart goniometer. Five μL aliquots of water were dropped onto the surface, and the image was recorded using the goniometer’s camera. The contact angle value was measured from the images using DROPimage software.

### Protein Binding Assay

The adsorption behavior of the blood-associated proteins albumin (Alb) and fibrinogen (Fib) onto the plane Ti, TiNT, and TiNT-TAN surfaces was examined using the previously reported technique.^40,58^ Before being used in the experiment, all surfaces were sterilized under UV radiation for 30 min and washed with sterile phosphate-buffered saline (PBS). These sterile surfaces were incubated in two 48-well plates containing 100 μg/mL of human fibrinogen and another containing 100 μg/mL of albumin at 37 °C for two h while being shaken at 100 rpm. After incubating for 2 h, the protein solution was aspirated and rinsed with PBS and water. Protein adsorption was investigated by measuring the percentage of nitrogen atom contribution by using an X-ray photoelectron spectroscopy (XPS) scan of different surfaces before and after protein binding.

### Separation of Human Platelet-Rich Plasma

Two healthy people consented to give blood by venous phlebotomy, following the National Institutes of Health’s “Guiding Principles for Ethical Research” guidelines and utilizing methods authorized by the Colorado State University Institutional Review Board. A phlebotomist took blood samples into 10 mL vacuum tubes coated with EDTA. Platelet-rich plasma (PRP) was obtained by centrifuging whole blood for 10 min at 150 g. The plasma containing platelets and leukocytes was removed and rested for 10 min before further use.

### Platelet Adhesion, Activation, and Aggregation on Different Surfaces^40,47^

Sterile Ti, TiNT, and TiNT-TAN surfaces were placed in a 48-well plate containing 500 μL of separated PRP. The plate was then incubated at 37 °C and 5% CO_2_ using a horizontal shaker operating at 100 rpm for 2 h. Following incubation, the plasma was removed, and the surfaces were rinsed with PBS to remove nonadherent or weakly adhered platelets.

Fluorescence and scanning electron microscopy (SEM) were used to evaluate platelet adhesion, activation, and aggregation on different surfaces. Surfaces for imaging under the fluorescence microscope were stained with a 2 μM calcein-AM solution in PBS for 30 min. ImageJ was used to measure the total area covered by platelets from the fluorescent images.

Surfaces used for SEM imaging were fixed with primary fixative (3.0% glutaraldehyde, 0.1 M sodium cacodylate, and 0.1 M sucrose) for 45 min, followed by a secondary fixation in a buffer solution containing the fixative components, except for the glutaraldehyde, for 10 min. The surfaces were then dehydrated using a series of ethanol solutions (35%, 50%, 70%, and 100%) with a 10 min incubation in each solution. All samples were sputter-coated with gold (10 nm) and imaged via SEM (JSM-6500F JEOL, Tokyo, Japan).

### Cell Viability, Adhesion, and Proliferation

#### Cell Culture

Human adipose-derived stem cells (ADSCs) were extracted from abdominal and thigh subcutaneous fat samples. This cell isolation was performed previously by Kimberly Cox-York in the Food Science and Human Nutrition Department at Colorado State University as part of an earlier study. The Colorado State University Institutional Review Board approved the protocol for obtaining ADSCs from healthy volunteers. ADSCs were cultured in a growth medium containing 90% MEM alpha modification (1×, Cytiva, Marlborough, MA, USA), 10% fetal bovine serum (FBS), and 1% penicillin–streptomycin.^42,43^ For all experiments, ADSCs below passage 5 were used.

#### Cytotoxicity Assay

A lactate dehydrogenase (LDH) cytotoxicity assay was performed (CyQUANT LDH cytotoxicity assay kit, ThermoFisher Scientific, Waltham, MA, USA) to assess the cytotoxicity of the surfaces.^42,43^ Before cell seeding, all surfaces were placed in a 48-well plate and sterilized under UV light for 30 min, followed by a PBS wash. ADSCs were then seeded directly onto the prepared surfaces at a 30,000 cells/mL concentration and incubated for 24 h at 37 °C in a 5% CO_2_ environment. Cells were cultured on polystyrene (PS) surfaces as a negative control, and for positive control, cells on polystyrene were treated with 1.0% Triton for 45 min. Following the 24 h incubation period, culture media from each well was extracted and combined with an equal volume of LDH substrate reagent solution (QuantiChrom BioAssay Systems, Hayward, CA, USA) in a 96-well plate. This mixture was then incubated for 30 min. Using a plate reader (FLUOstar Omega, BMG LABTECH, Cary, NC, USA), the absorbance of the LDH solution in each well was measured at wavelengths of 490 and 680 nm. The experiment utilized five replicates for each surface type (Ti, TiNT, and TiNT-TAN).

#### Cell Viability Assay

The cell viability on Ti, TiNT, and TiNT-TAN surfaces was assessed using an Alamar Blue assay from Invitrogen. 15000/mL cells were seeded in a 24-well plate on different surfaces. After 4 and 7 days of cell culture, the substrates were incubated in culture media with 10% of assay reagent at 37 °C for 6 h. After this incubation period, the absorbance of the resulting solutions was measured at 570 and 600 nm by a plate reader. The Alamar Blue reduction percentage correlates with the activity of viable cells and was calculated as described by the manufacturer protocol. ADSCs were also cultured in empty wells (positive control), while media without cells served as a negative control.^42^

### Cell Adhesion and Proliferation of Different Surfaces^42^

After 4 and 7 days, the cells found adhering to the substrates were fixed by incubation in a 3.7% formaldehyde solution in PBS for 15 min, with three subsequent rinses with PBS. The cells were then permeabilized in a 1% Triton X-100 solution in PBS for 3 min, followed by a 2-fold rinse with PBS. After that, the substrates were placed in rhodamine phalloidin solution (70 nM, Cytoskeleton) for 20 min, followed by 5 min incubation in DAPI stain solution (300 nM, ThermoFisher Scientific). After being rinsed with PBS, the substrates were imaged using a fluorescence microscope (ZEISS). The number of cells on the surface was obtained by counting the stained nuclei.

### Statistical Evaluation

For each sample category, experiments were performed with at least three independent replicates. Data are presented as mean values accompanied by their corresponding standard deviations. To determine statistical significance, we employed a two-tailed unpaired *t*-test. *P*-values were calculated, with statistical significance defined as ****p < 0.0001, ***p < 0.001, **p < 0.01, and *p < 0.05.

## Results and Discussion

### Fabrication of Titania Nanotubes and Characterization

**Figure 1:**
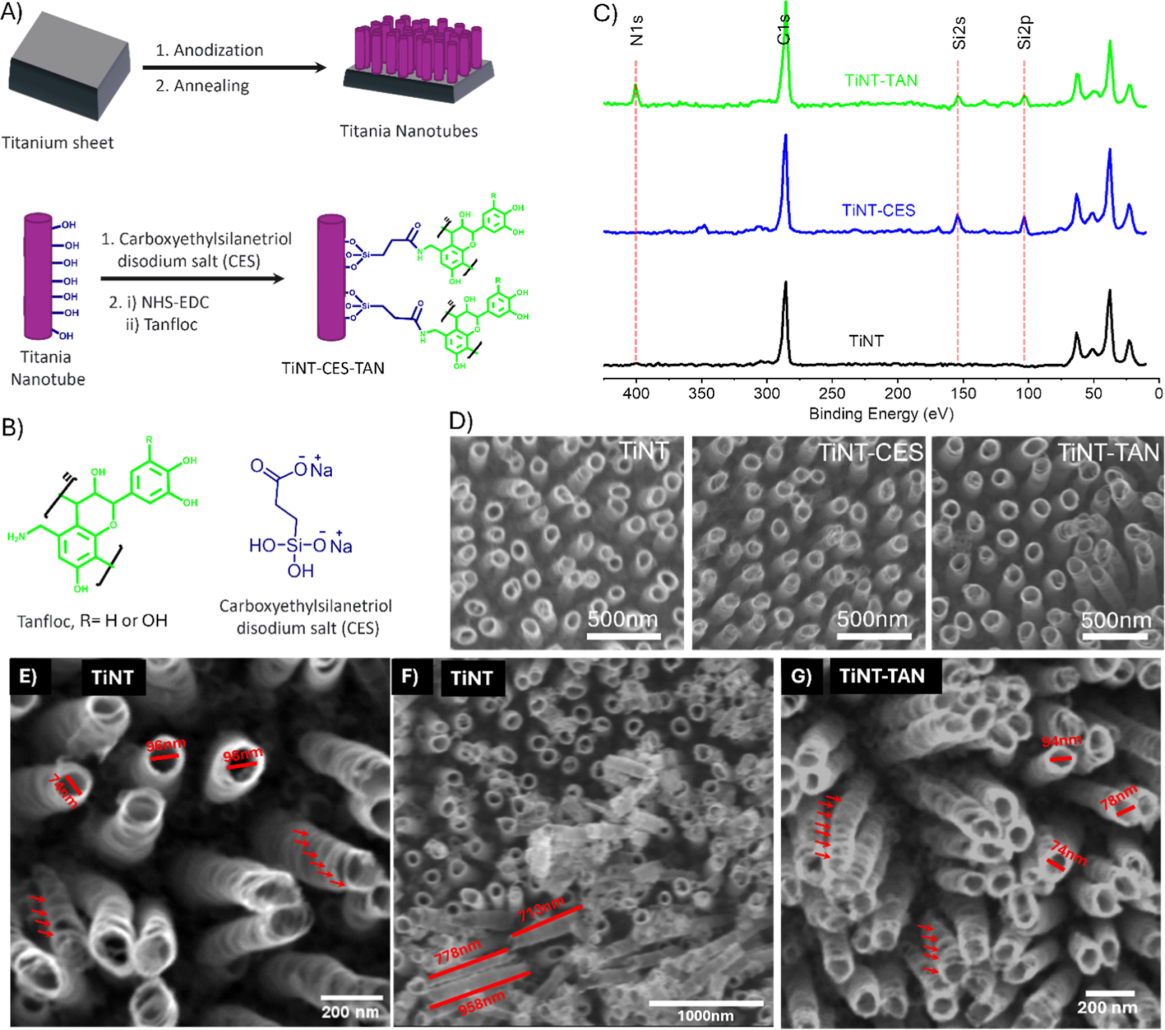
(A) Schematic illustration of the titania nanotube synthesis on the titanium surface followed by covalent conjugation of Tanfloc. (B) Chemical structures of Tanfloc and carboxyethylsilanetriol employed in the conjugation process. (C) Comparative XPS spectra of TiNT, TiNT-CES, and TiNT-TAN surfaces demonstrating successful Tanfloc conjugation. (D) SEM micrographs showing overall morphology were preserved following Tanfloc functionalization on various surfaces. (E,F) SEM images of TiNT and (G) SEM image of TiNT-TAN showing dimensional measurement.

An electrochemical anodization procedure was used to fabricate titania nanotube (TiNTs) arrays on titanium surfaces, followed by 3 h of thermal annealing at 530 °C with a 15 °C/min temperature increase in an ambient oxygen environment (Figure 1A).^31,43−47^ The formation of TiNTs was confirmed by scanning electron microscopy (SEM) (Figure 1D). SEM images revealed that the stacking of the nanoring forms the nanotubes and seems like a spring. SEM image analysis using ImageJ showed that the nanotubes exhibited a diameter ranging between 70 and 100 nm, with lengths spanning from 700 to 1000 nm, and the ring thickness is approximately 15–20 nm (Figure 1E,F). After the synthesis of TiNTs, Tanfloc was covalently conjugated on the surface by carboxylation using carboxyethyl silane triol (CES) conjugation,^48,49^ followed by amide coupling,^50,51^ as previously reported (Figure 1 A).^43^ The conjugation of Tanfloc was confirmed through X-ray photoelectron spectroscopy (XPS) (Figure 1C).^43,52^ The appearance of silicon peaks in XPS of TiNT-CES at 103.1 eV for Si 2p and 154.3 eV for Si 2s (Figure 1C, blue spectra), which are absent in XPS of TiNT (Figure 1C, black spectra), indicates the attachment of CES. Further, the nitrogen (N 1s) peak at 400.7 eV and the silicone peaks in XPS of TiNT-TAN (Figure 1C, green spectra) confirmed the conjugation of titania.^43,52,53^

Further, in the FT-IR spectra of TiNT-TAN (Figure S1 Supporting Information), a broad band of phenolic –OH and amine groups around 3323 cm^–1^ and a weak shoulder with this broad band in the C–H band region for aliphatic and aromatic C–H around 2954 cm^–1^ appeared. The shoulders at 806 and 606 cm^–1^ indicate the metallic oxide Si–O bonds along with Ti–O bonds. Additionally, the band at the carbonyl region 1649 cm^–1^ indicates the formation of amide bonds and confirms the conjugation of TAN. The SEM images of TiNT, TiNT-CES, and TiNT-TAN showed that the structural morphology is almost identical for functionalized and nonfunctionalized titania nanotube surfaces (Figure 1D,G). To evaluate the stability of TiNT-TAN surfaces, researchers conducted an immersion test in PBS at 35 °C followed by XPS analysis (Supporting Information). The XPS survey, spanning 3 weeks, demonstrated that the principal elemental peaks, notably silicon and nitrogen, remained predominantly unchanged. These results indicate that the Tanfloc maintains a strong adherence to the titania nanotube surface, evidencing a robust and enduring bond.

### Contact Angle Measurement

Surface wettability significantly influences the biological response to implanted materials by affecting protein adsorption, platelet adhesion, and cellular behavior, including adhesion, proliferation, and differentiation.^31,54^ The water contact angle for the plain Ti surface was around 60°, indicating the Ti surface’s hydrophobic nature. TiNT and TiNT-TAN demonstrate hydrophilic properties with contact angles of less than 10°.^31^ TiNT surfaces have hydrophilicity due to nanotube arrays, which have remained unaffected on TiNT-TAN surfaces. These hydrophilic surfaces are generally known to enhance cellular adhesion and differentiation.^31,54,55^ Hydrophilic TiNT surfaces typically adsorb less protein than Ti surfaces due to repulsive solvation forces from bound water molecules.^31,54,55^

### Protein Adsorption Assay

**Figure 2:**
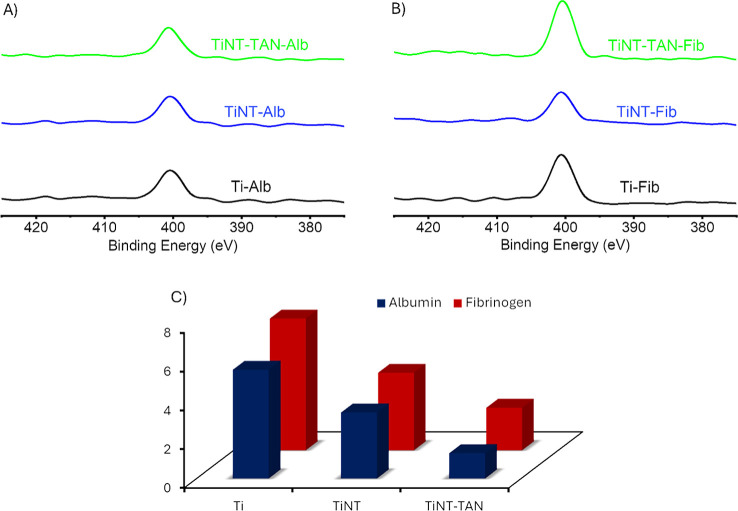
XPS survey spectra of different surfaces Ti, TiNT, and TiNT-TAN: (A) after albumin (Alb) binding and (B) after fibrinogen (Fib) binding. (C) The bar graph represents the net percentage of nitrogen on different surfaces Ti, TiNT, and TiNT-TAN after treatment with fibrinogen and albumin proteins.

Blood proteins quickly interact and adsorb onto the biomaterial surface based on surface properties and form a primary protein layer.^56,57^ These protein layers subsequently influence platelets and blood behavior on the surface.^56,57^ Therefore, a protein binding experiment was conducted with two significant blood proteins: albumin and fibrinogen on the different surfaces.^47^ Albumin, an abundant component in blood serum, rapidly interacts with biomaterial surfaces and reduces platelet adhesion in some instances, potentially inhibiting blood clotting.^47,57,58^ On the other hand, fibrinogen adsorption triggers the intrinsic pathway of blood coagulation, playing a crucial role in clot formation.^59^ Higher levels of adsorbed fibrinogen generally accelerate blood clot formation and may induce thrombosis in some cases.^47,57,58^ Controlling protein adsorption, mainly fibrinogen and albumin, is crucial to the improved biocompatibility and performance in medical devices. The surfaces treated with albumin and fibrinogen were then analyzed using X-ray photoelectron spectroscopy (XPS) (Figure 2A,B),^40^ and the percentage nitrogen (N 1s) contribution was calculated from survey spectra of different surfaces before and after protein binding (Table 1).^40,58^ XPS survey spectra revealed distinct relative atomic weight percentages of N 1s content across the surfaces before and after protein binding (Table 1 and Figure 2A,B). Before protein exposure, Ti and TiNT surfaces showed no nitrogen content, while TiNT-TAN showed 4% nitrogen due to the presence of Tanfloc (Table 1). XPS survey analysis after protein binding revealed that the Ti surface binds the highest amount of albumin, followed by the TiNT surface, and the TiNT-TAN binds the lowest amount of albumin as net % N 1s content decreases (Table 1). Although fibrinogen consistently showed higher adsorption levels than albumin across all surfaces, the same trend is obtained for fibrinogen adsorption with higher adsorption on Ti, followed by TiNT and TiNT-TAN (Table 1 and Figure 2C). The XPS results showed that the presence of Tanfloc on TiNT-TAN surfaces reduces the binding of proteins compared to Ti and TiNT surfaces. Possibly, the zwitterionic-like moiety in Tanfloc reduces the absorbance of proteins.^60,61^

**Table 1:** Atomic Percentage Nitrogen (N 1s) before and after Fibrinogen and Albumin Binding Calculated from the XPS Survey Using MultiPak Software

surface	% N 1s before protein binding	% N 1s after protein binding		net % N 1s adsorbed after protein binding	
		albumin	fibrinogen	albumin	fibrinogen
Ti	0	5.6	6.8	5.6	6.8
TiNT	0	3.4	4	3.4	4
TiNT-TAN	4	5.3	6.2	1.3	2.2

### Platelet Adherence and Activation

**Figure 3:**
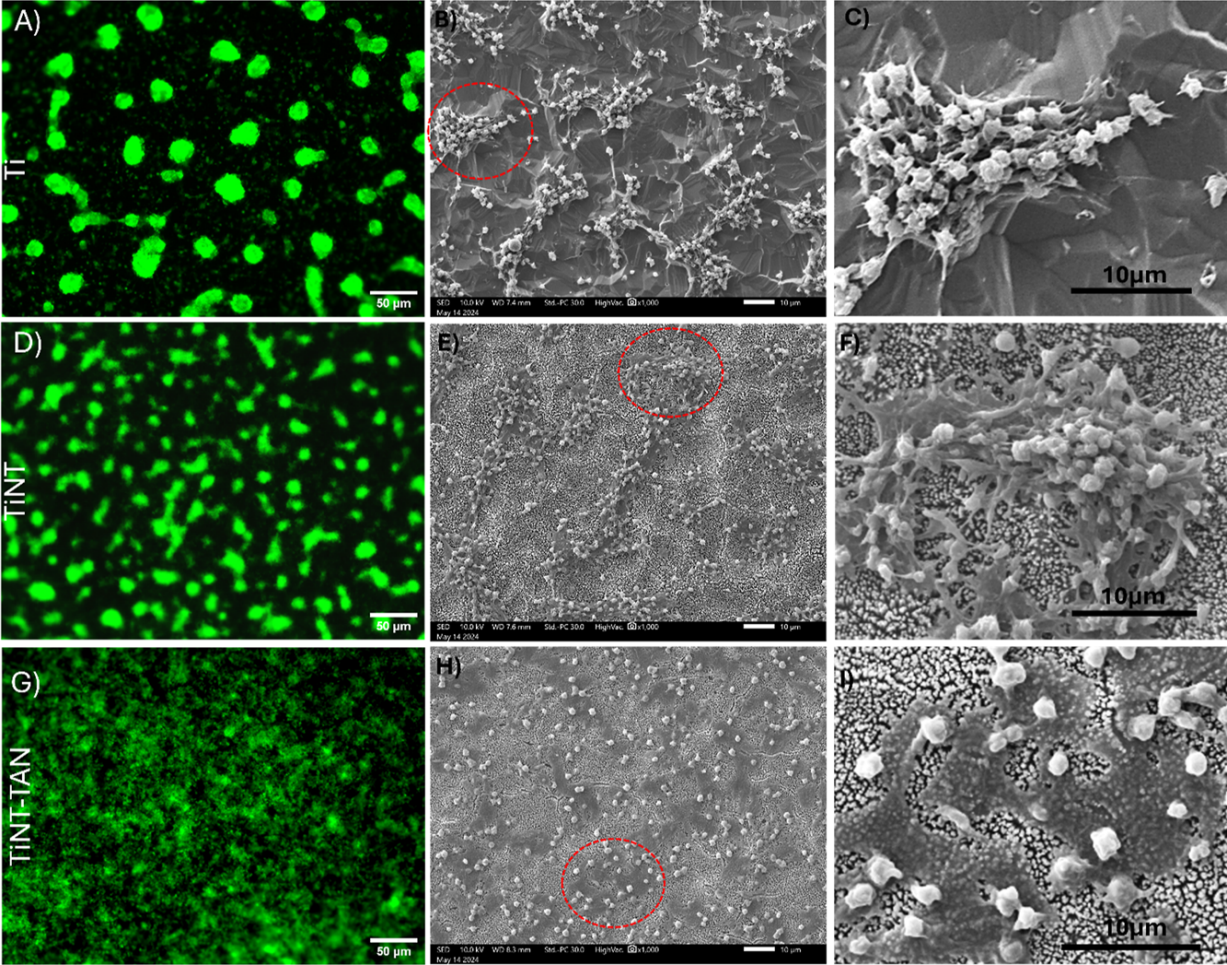
Platelet adhesion and morphology on various surfaces after 2 h incubation in platelet-rich plasma (PRP). Left column: Fluorescence microscopy images (green) showing platelet adhesion on (A) Ti, (D) TiNT, and (G) TiNT-TAN surfaces. Right columns: Corresponding scanning electron microscopy (SEM) images illustrating platelet morphology and arrangement. (B) Platelets on unmodified titanium, with (C) providing a higher magnification view of the area circled in red. (E) Platelets on the TiNT surface, with (F) offering a close-up of the highlighted region. (H) Platelets on the TiNT-TAN surface, with (I) presenting a magnified view of the selected area.

The reduction of fibrinogen adsorption resulted in a reduced platelet adhesion on the TiNT-TAN surface. Ti, TiNT, and TiNT surfaces were incubated in platelet-rich plasma for 2 h at 37 °C to investigate platelet behavior. These incubated surfaces were then analyzed by fluorescence microscopy and SEM.^8,40,58^ Fluorescence images of calcein-AM-stained platelets showed different adsorption patterns on different surfaces (Figure 3). The image of the Ti surface showed distributed bright spots (Figure 3A). In contrast, TiNT showed smaller deformation (elongated) of these spots of adhered platelets (Figure 3D). On the other hand, the fluorescence image of TiNT-TAN showed platelets spreading evenly instead of bright spots (Figure 3G). These results indicate that TiNT-TAN interacted with platelets differently, supporting the protein adsorption experiment results. A quantification analysis of the fluorescence image was performed using ImageJ software to evaluate the area of surface covered by adhered platelets on the different surfaces (Figure S2 Supporting Information). Results revealed that platelets covered the maximum area on the TiNT-TAN surfaces rather than the Ti and TiNT surfaces. Further, SEM images were taken and analyzed for closer insight into understanding the platelet behavior on these surfaces. SEM images revealed that the brighter and bigger spots in the fluorescence image are aggregates of platelets adhered to the titanium surface (Figure 3B,C). These platelets exhibited pseudopod formation, extending outward and connecting with one another, leading to localized platelet accumulation. In the case of TiNT surfaces, further progress in platelet activation has appeared, with more extensive pseudopod development and some flattening of platelets (Figure 3E,F). However, the overall aggregation pattern is similar to that observed on the Ti surfaces. Interestingly, the TiNT-TAN surface produced a distinct platelet response. SEM reveals that TiNT-TAN surface platelets displayed lower adherence but accelerated activation without aggregation (Figure 3H,I). Platelets transformed into a flattened morphology spread extensively across the surface, forming a layer over the TiNT-TAN surface (Figure 3I). The formation of a protective layer of platelets corresponds well to the fluorescence results. This reduction of platelet aggregation can help reduce the risk of thrombosis formation.^8,62^ Additionally, the ability of Tanfloc surface coatings to form a protective layer can be beneficial in blocking bleeding, wound healing, cellular growth, and tissue engineering.^10−12^

### Cytotoxicity and Cell Viability

**Figure 4:**
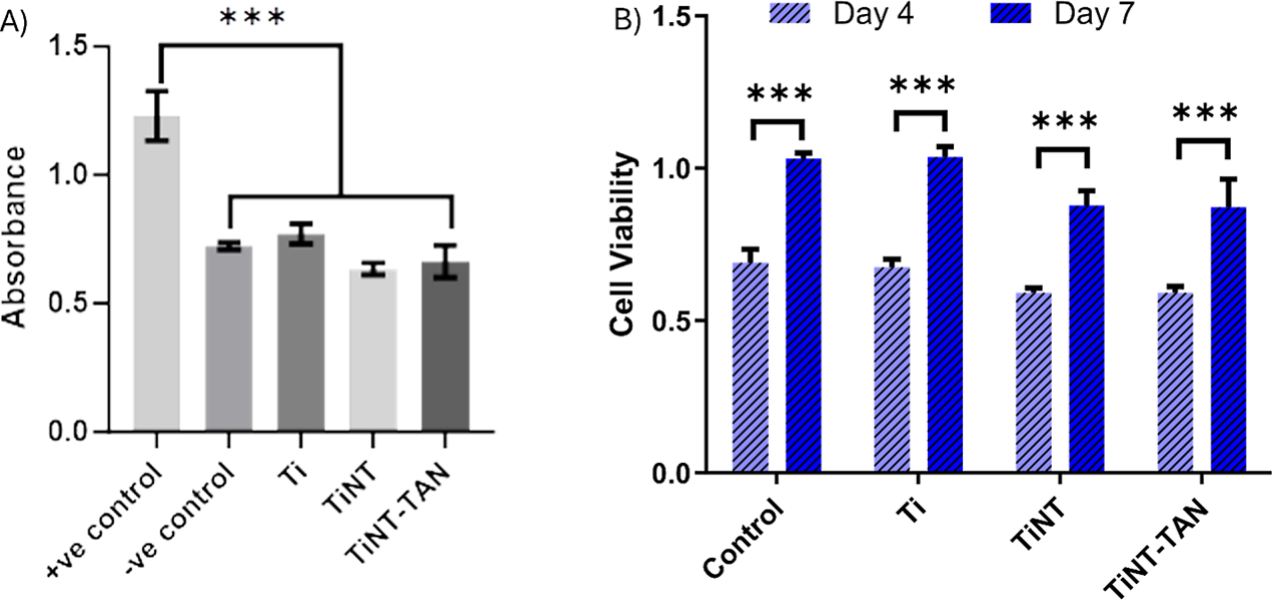
(A) LDH assay results, representing the noncytotoxicity of different surfaces. Significance: *** *p*-value <0.001. (B) Cell viability of ADSCs grown on different surfaces was measured using an Alamar blue assay. Significance: ***p-value <0.001.

Medical devices’ long-term dependability and effectiveness are contingent upon their compatibility with biological systems. Minimizing adverse reactions, facilitating a smooth integration with the surrounding host environment, and prolonging the functional lifespan of these medical devices all depend on biomaterials being compatible with living tissues. Therefore, cytotoxicity, cellular viability, and proliferation experiments were performed to evaluate this compatibility. An LDH assay was performed to assess these surfaces’ cytotoxicity.^43^ The quantification of the release of the LDH enzyme from damaged cells is widely used for cytotoxicity tests. ADSCs were cultured directly on Ti, TiNT, and TiNT-TAN for 24 h to assess the cytotoxicity.^43^ The LDH assay results demonstrated that there was no cytotoxicity of the titanium, TiNT, or TiNT-TAN surfaces, with the released LDH in the media for all samples being nearly identical to the negative control, which was much lower than that of the positive control (Figure 4A). Further, an Alamar blue assay was performed to evaluate cellular viability on these surfaces after 4 and 7 days of ADSC culture.^42^ This assay relies on the ability of metabolically active cells to reduce resazurin, a component of the Alamar Blue reagent, to resorufin through the action of dehydrogenase enzymes (Figure 4B). A greater reduction of Alamar Blue indicates a higher number of viable cells on the substrates.^42^ There were no significant changes in the viability of the ADSCs on different samples. However, a notable increase in cell growth from day 4 to day 7 on all substrates was observed (Figure 4B). This suggests that ADSCs maintain a comparable viability across all surfaces, which aligns well with cytotoxicity test results.

### Cell Adherence and Proliferation

**Figure 5:**
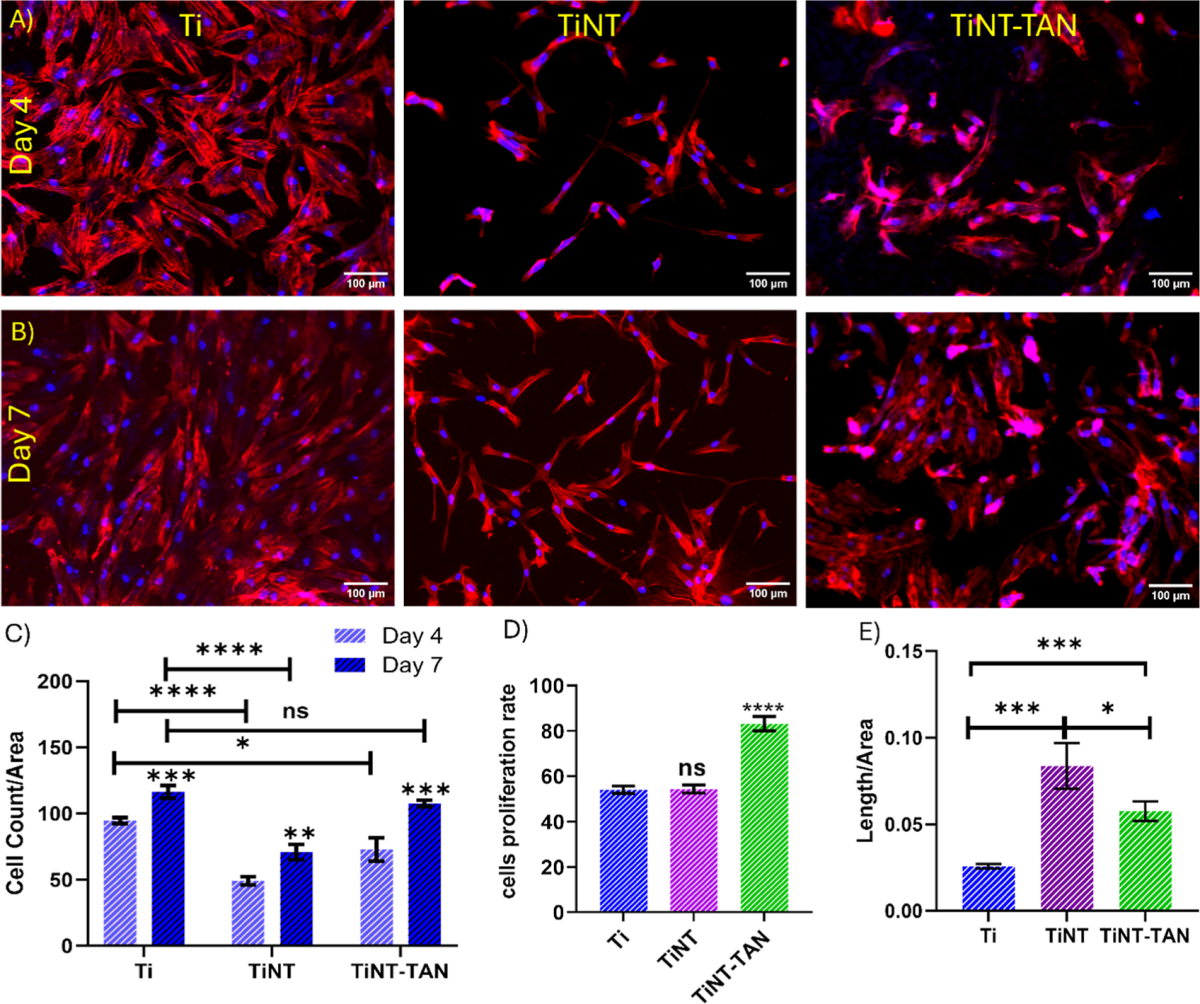
Panels (A,B) show representative fluorescence images of ADSCs stained with DAPI and rhodamine–phalloidin, adhered on Ti, TiNT, and TiNT-TAN surfaces. Panel (A): after 4 days of cell cultures; Panel (B): after 7 days of cell cultures. (C) Cell counts adhered on different surfaces stained with DAPI corresponding to panels (A,B) (the *p*-values indicated over error bars represent a comparison over the same surface from day 4 to day 7); (D) rate of cell proliferation from day 4 to day 7. (E) Cell length-to-area ratio showing quantification of elongation of cells on different surfaces on day 7 of culture. Error bars indicate the mean with s.d. and the statistical significance (*p*-value) obtained using a two-tailed unpaired *t*-test, with the following levels of relevance: ****p < 0.0001, ***p < 0.001, **p < 0.01, and *p < 0.05.

The early phases of cell attachment and proliferation are critical in determining how well a medical device integrates with the surrounding host tissues and the device’s long-term stability. Fluorescence microscopy was used after 4 and 7 days of cell cultures to visualize the adherence and growth of cells on different surfaces (Figure 5A,C). After 4 days of culture, fluorescence imaging revealed that the titanium surface was populated with healthy, flattened cells. In contrast, the TiNT surface exhibited an elongated cellular morphology. The population of cells is less on the TiNT and TiNT-TAN surfaces than on the Ti surface. The TiNT-TAN surface showed cells similar to those on TiNT (Figure 5A). Cell morphology analysis using ImageJ revealed significant differences in elongation across various surfaces. The length-to-area ratio was used as a metric to quantify cell elongation. Results showed that cells cultured on the TiNT surface exhibited a more pronounced elongated shape in comparison to those on plain Ti surfaces. Interestingly, cells on the TiNT-TAN surface displayed an intermediate level of elongation, less pronounced than that on TiNT but still more elongated than that on Ti. This comparative analysis highlights that the nanostructured surfaces promote cell elongation. The elongation of cells indicates the ability to achieve osteoblast differentiation and bone-forming capacity. Elongated cell shapes on orthopedic implant surfaces promote superior bone integration through multiple pathways.^63^ This morphology enhances osteogenic differentiation and signaling, increases cytoskeletal tension and focal adhesion formation, and boosts alkaline phosphatase activity.^63,64^ These factors collectively contribute to improved bone formation, stronger bonding between the medical device and surrounding tissue, and enhanced osteoblast differentiation, ultimately leading to more effective implant osseointegration.^63,64^ After 7 days of cell culture, growth was evident on all surfaces, although with distinct patterns. The titanium surface displayed densely adhered cells, while the TiNT surface showed more elongated cellular structures. The TiNT-TAN surface exhibited denser and flatter cell growth than did TiNT (Figure 5B). Cells adhered to different surfaces were quantified by counting DAPI-stained nuclei. The quantification revealed that after 7 days of culture, the number of cells on TiNT-TAN was almost similar to that on Ti’s surface (Figure 5C). Additionally, the rate of cell proliferation from day 4 to day 7 showed a faster growth of cells on TiNT-TAN surfaces (Figure 5D). The outcomes of the noncytotoxicity, cellular viability, cell adhesion, and proliferation rate on the TiNT-TAN surface demonstrate the combined effects of nanotube arrays and Tanfloc functionality. The nanostructured environment increases the surface area for cell attachment, and different functional groups on Tanfloc facilitate faster integration with cells through various noncovalent interactions.^63^ Additionally, the hydrophilic nature of the TiNT-TAN surface further supported cellular attachment and integration.

Low fibrinogen adsorption and strong platelet activation on TiNT-TAN surfaces contradict each other, indicating that Tanfloc directly influences platelet behavior on the titania surface rather than acting through fibrinogen.^59^ Tanfloc is an amphoteric polymer and exhibits zwitterionic-like characteristics, functioning as both a polycation and a polyanion with charge-neutralization capabilities. Zwitterionic property is crucial for biocompatibility as it contributes to forming a hydration layer on the surface, which can significantly reduce nonspecific protein adsorption.^61,65^ The balanced charge distribution mimics the outer surface of cell membranes, allowing for better integration.^61^ For example, a zwitterionic polymer poly(2-methacryloyloxyethyl phosphorylcholine) generally prevents protein adsorption by forming a hydration layer around the positive and negative charges.^60,61^ The balance between cationic and anionic groups could create a surface microenvironment that mimics physiological conditions, potentially explaining the controlled platelet activation observed in our study.^61,65^ The spreading and flattening of platelets on the TiNT-TAN surface without unwanted platelet aggregation indicate a controlled activation. Unlike traditional zwitterionic polymers that aim for complete inhibition, Tanfloc may allow for more distinct and controlled interactions with blood proteins and platelets.^60,61^ This may be due to the presence of multiple functional groups like phenolic, amine, and aromatic rings and cationic and anionic nature that can interact differently with various protein components.^39,43^ The positively charged moieties could interact with negatively charged regions on the platelet membrane such as phosphatidylserine residues.^61,66^ This electrostatic interaction might influence the initial adhesion of platelets to the surface and subsequent activation processes. Furthermore, polyphenolic and other functional groups in the Tanfloc polymer can interact with platelet membrane proteins, including integrins and glycoproteins, through hydrogen bonding.^39,67,68^ Such interactions may modulate the conformational changes in these proteins, affecting their signaling capabilities and, thus, influencing platelet activation and aggregation pathways.

**Figure 6:**
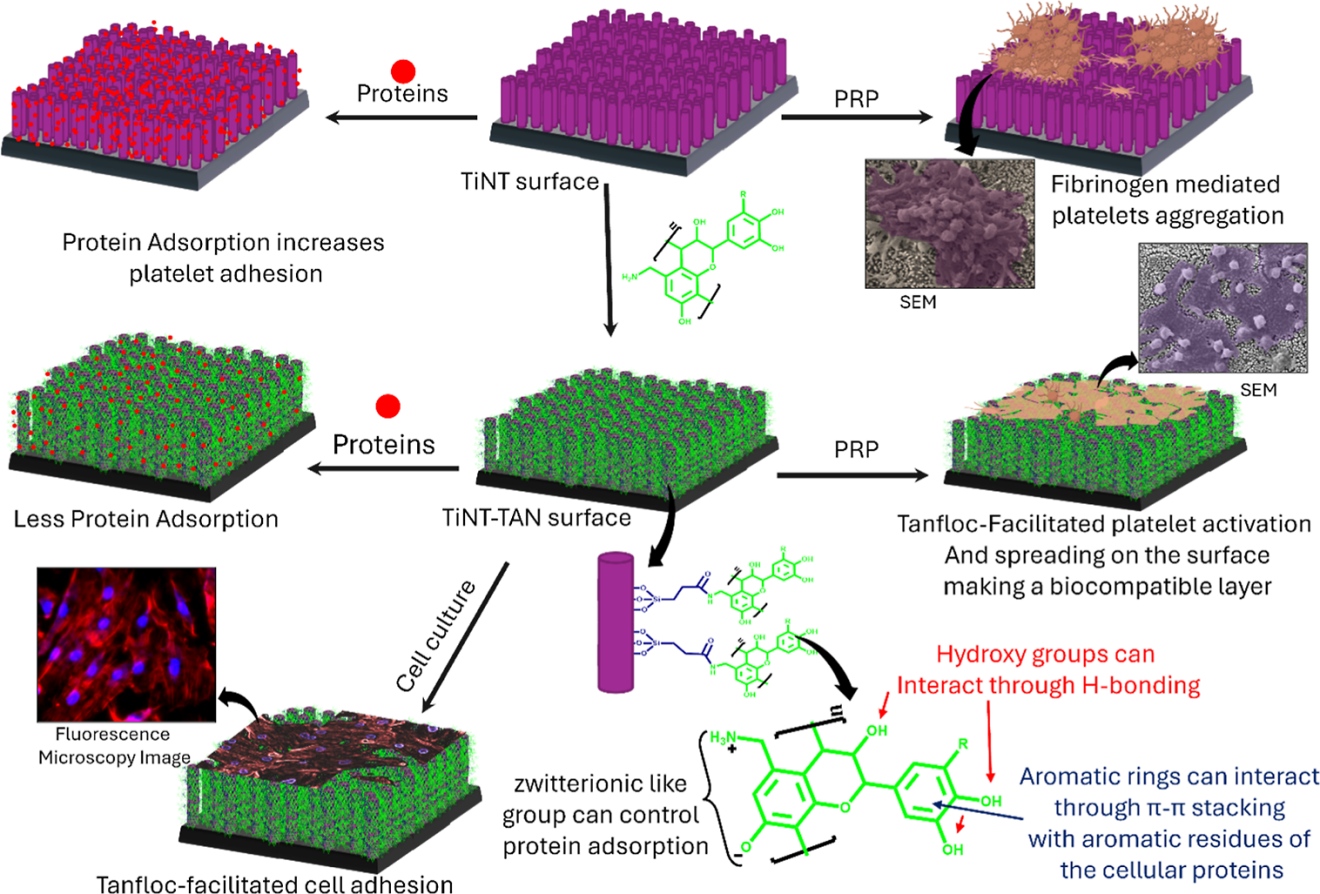
Conceptual representation of TiNT-TAN surface functionality. This illustration showcases the combined effects of nanotubular arrays and covalently attached Tanfloc. Tanfloc’s versatile properties enhance the titania surface’s biocompatibility, promoting optimal protein adsorption, controlled platelet activation, and improved cellular adhesion. The potential noncovalent interactions of Tanfloc contribute to regulated adherence, further optimizing the surface’s biological performance.

It is essential to carefully control platelet adhesion and activation, which is crucial in determining the biocompatibility of implanted materials.^62^ Cellular adherence and proliferation further support the efficacy and acceptance of the TiNT-TAN biocompatibility. Figure 6 is a schematic representation of these findings. The nanotube structures promote the elongation of cells,^31,63^ and Tanfloc provides a more biocompatible environment that facilitates strong attachment and a higher cell proliferation rate. The current results, together with the previous findings that demonstrated the potent antibacterial and antibiofilm capabilities of TiNT-TAN, make it a potentially multifunctional biomaterial for medical devices.^42,43^

## Conclusions

This study investigated the interaction of TiNT-TAN surface with blood components, proteins and platelets, cell adhesion, and cell proliferation. The experimental results revealed that the adsorption of blood proteins albumin and fibrinogen is reduced on TiNT-TAN surfaces more than on TiNT surfaces. Platelets uniquely interacted with Tanfloc-functionalized surfaces and displayed controlled adhesion and activation. The platelets on the TiNT-TAN surface get flattened in contrast to aggregation on Ti and TiNT surfaces and significantly (****p < 0.0001) cover the TiNT-TAN surface. Perhaps this is due to the zwitterionic-like nature of Tanfloc, which inhibits protein adsorption and platelet aggregation through electrostatic interactions. Meanwhile, other organic functional groups on Tanfloc, such as phenolic, amine, and aromatic rings, may facilitate platelet spreading through noncovalent interaction with the platelet membrane. Additionally, TiNT-TAN surfaces demonstrated favorable cellular viability and improved proliferation (****p < 0.0001) without any cytotoxicity.

## Supporting Information Available

FT-IR spectra, quantification of platelets adhered, and XPS survey spectra (PDF)
