# Supplementary material for: Tanfloc-Modified Titanium Surfaces: Optimizing Blood Coagulant Activity and Stem Cell Compatibility
Source: ACS Biomater Sci Eng. 2025 Feb 27;11(3):1445–55. doi: 10.1021/acsbiomaterials.4c02106 (PMC11897940; doi:10.1021/acsbiomaterials.4c02106)
Supplement: Supplementary file 1 — ab4c02106_si_001.pdf [file ab4c02106_si_001.pdf]

Tanfloc-Modified Titanium Surfaces: Optimizing Blood Coagulant Activity and Stem Cell Compatibility

Ramesh Singh[a], Liszt Y. C. Madruga[a], Aniruddha Savargaonkar[b], Alessandro F. Martins[c,d], Matt J. Kipper[c], Ketul C. Popat[a, d, *]

[a]Department of Bioengineering, College of Engineering and Computing, George Mason University, VA, USA.

[b] Department of Mechanical Engineering, Colorado State University, CO, USA.

[c] Department of Chemical and Biological Engineering, Colorado State University, CO, USA.

[d] Department of Chemistry, Pittsburg State University, Pittsburg, KS, USA.

* Author of correspondence: ketul.popat@colostate.edu and kpopat@gmu.edu

Figures:

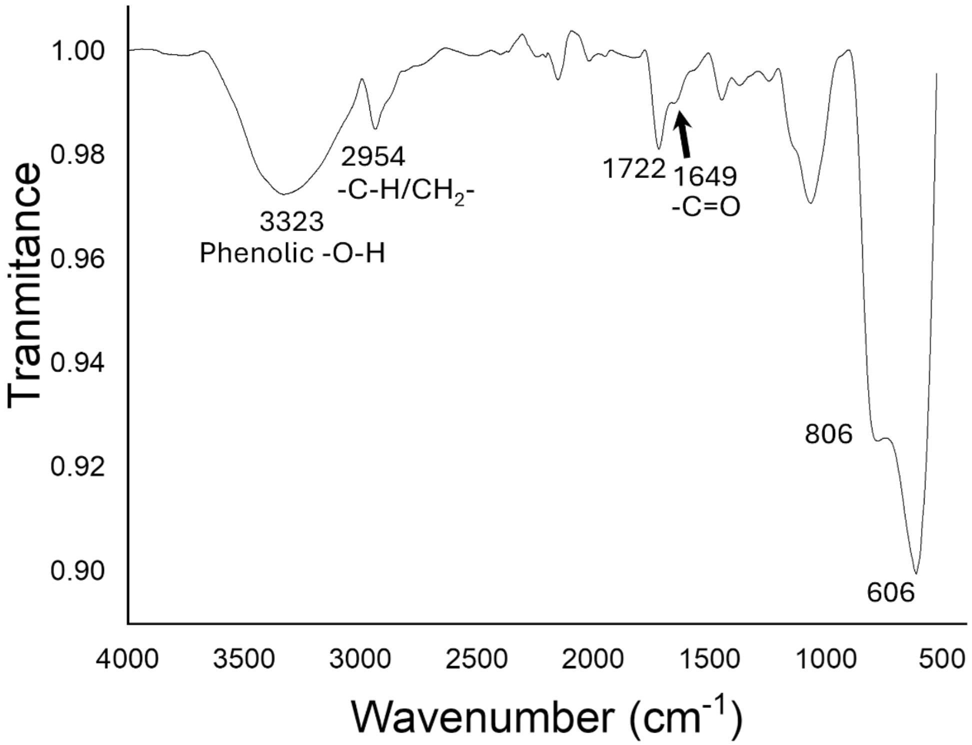

Figure S1: FT-IR spectra of TiNT-TAN

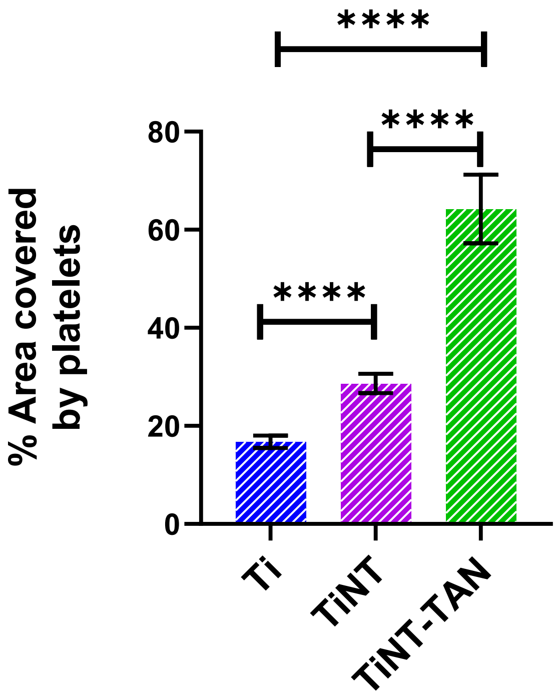

Figure S2: Quantification of platelets adhered on different surfaces corresponding to the fluorescence images in Figure 3.

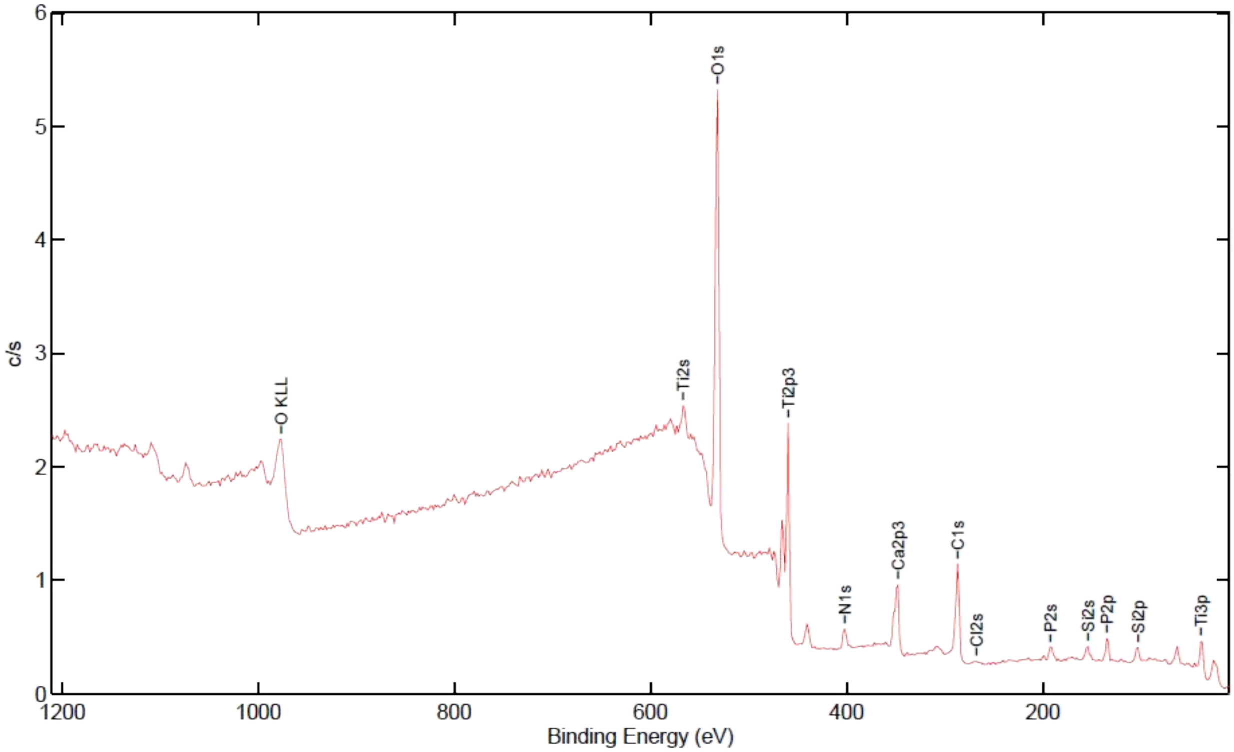

Figure S3: XPS survey spectra of TiNT-TAN surfaces after 3 weeks of PBS incubation. The presence of all peaks in both spectra demonstrates the durable adhesion of TAN to the titania nanotubes.
